# Assessing model adequacy for Bayesian Skyline plots using posterior predictive simulation

**DOI:** 10.1371/journal.pone.0269438

**Published:** 2022-07-25

**Authors:** Emanuel M. Fonseca, Drew J. Duckett, Filipe G. Almeida, Megan L. Smith, Maria Tereza C. Thomé, Bryan C. Carstens

**Affiliations:** 1 Department of Evolution, Ecology and Organismal Biology, The Ohio State University, Columbus, OH, United States of America; 2 Museum of Biological Diversity, The Ohio State University, Columbus, OH, United States of America; 3 Department of Zoology, Federal University at Juiz de Fora, Juiz de Fora, Minas Gerais, Brazil; 4 Department of Biology and Department of Computer Science, Indiana University, Bloomington, IN, United States of America; Universitat Pompeu Fabra, SPAIN

## Abstract

Bayesian skyline plots (BSPs) are a useful tool for making inferences about demographic history. For example, researchers typically apply BSPs to test hypotheses regarding how climate changes have influenced intraspecific genetic diversity over time. Like any method, BSP has assumptions that may be violated in some empirical systems (e.g., the absence of population genetic structure), and the naïve analysis of data collected from these systems may lead to spurious results. To address these issues, we introduce P2C2M.Skyline, an R package designed to assess model adequacy for BSPs using posterior predictive simulation. P2C2M.Skyline uses a phylogenetic tree and the log file output from Bayesian Skyline analyses to simulate posterior predictive datasets and then compares this null distribution to statistics calculated from the empirical data to check for model violations. P2C2M.Skyline was able to correctly identify model violations when simulated datasets were generated assuming genetic structure, which is a clear violation of BSP model assumptions. Conversely, P2C2M.Skyline showed low rates of false positives when models were simulated under the BSP model. We also evaluate the P2C2M.Skyline performance in empirical systems, where we detected model violations when DNA sequences from multiple populations were lumped together. P2C2M.Skyline represents a user-friendly and computationally efficient resource for researchers aiming to make inferences from BSP.

## Introduction

Posterior predictive simulation (PPS) is a commonly used technique for assessing model adequacy in a Bayesian framework [1]. PPS samples parameter values from the posterior distribution of an empirical analysis and simulates data that match the underlying assumptions of the model used to analyze the data. The probability distribution of the simulated data given the model is then compared to the actual data in order to assess model adequacy, either directly or via the use of proxy summary statistics. In either case, empirical data that are consistent with the assumptions of the model used to analyze the data will have probabilities distribution that are similar to the simulated data, and such a result would strengthen the confidence of the researcher in the inferences that result from the analysis of the empirical data. On the other hand, finding that the posterior distribution of the empirical data differs substantially from the posterior predictive distribution provides strong evidence that one or more of the underlying model assumptions has been violated. In sum, PPS may allow researchers to learn how a model does not fit the data [2, 3] providing perhaps the best approach to evaluating model adequacy for complex models [4].

Posterior predictive checks were introduced to molecular systematics by Huelsenbeck et al. [5] in the context of assessing the adequacy of models of sequence evolution, which are essential to the calculation of the posterior distribution in Bayesian inference. Work on assessing the fit of sequence evolution models has continued, with recent authors introducing posterior predictive approaches to evaluating the fit of models of sequence evolution in Bayesian phylogenetic inference [6], and the development of new statistics for detecting cases where model misspecification negatively influences phylogeny estimation [7]. PPS is gaining popularity for analyses conducted at the species level; for example it has been implemented to show that two common population genetic models perform poorly in describing the history of a duck species [8], has been used to identify instances of introgressive hybridization [9], and has been used to explore the accuracy of DNA barcoding efforts [10]. Recently, Duchene et al. [11] introduced a new software to assess the adequacy of phylodynamic models in infectious diseases investigations. Thus, posterior predictive assessments of model adequacy have the potential to improve investigations by verifying that the data collected from empirical systems are adequate to address the research questions. This can be particularly important when data are recycled or repurposed, that is, downloaded from public databases and used to address new questions (e.g., [12–14]).

There are millions of sequences available from ‘first generation’ phylogeographic investigations (e.g., BOLD, GenBank). These mitochondrial or chloroplast phylogeographic data sets are also still being collected by researchers, often as a first pass at data analysis in empirical systems (e.g., [15]). Such data are often used in multispecies comparative analyses, such as those investigating simultaneous divergence (e.g., [16, 17]) or expansion [13, 18] using hierarchical ABC. Similarly, these data can be used in automated phylogeography [19] and predictive phylogeography [20, 21]. However, each of these analyses makes certain assumptions about these data that may be difficult to assess. Hence, a practical limit on the repurposing of phylogeographic data is present when researchers cannot easily assess model adequacy.

Ideally, any phylogeographic data should be assessed by scientists in a manner that considers the model assumptions of the analyses that they plan to conduct. One example is the requirement of some analytical methods that the samples are free of population genetic structure, which can confound hierarchical tests of population expansion. To address this question, researchers have applied a species delimitation approach to identify structure within nominal species [18, 22]. Recently, Fonseca et al. [23] introduced a posterior predictive test of the data given the Generalized Mixed Yule Coalescent (GMYC) model in order to assess model adequacy, showing that large population sizes are likely to confound GMYC analysis. Here, we develop a posterior predictive test of model adequacy for population size changes using PPS on Bayesian Skyline plots (BSP; [24]).

Bayesian Skyline plots are a class of skyline-plot methods devised to infer the demographic history from DNA sequences using coalescent theory and co-estimation of genealogies and nucleotide substitution-parameters [25]. Introduced by Drummond et al. [24], BSPs are an extension of earlier Skyline-plot methods [26, 27] that enable phylogenetic uncertainty to be incorporated. To reconstruct the effective population size through time, BSPs estimate gene genealogies from a DNA alignment and simultaneously infer the demographic history from the gene genealogies. The coalescent model used in BSPs contains inherent assumptions about DNA sequences used in the analysis, notably that these were randomly sampled from a panmictic population and that the sequences are orthologous, nonrecombining, and neutrally evolving [25]. Because many datasets can potentially violate the first assumption (i.e., absence of genetic structure), we built an R package to assess the model adequacy for BSPs using PPS that can easily be incorporated into analysis pipelines.

### Demographic history in empirical datasets

Duchene et al. [11] advocated that empiricists explore the model adequacy of the skyline plot model as part of the inference process. To support this suggestion, we include an investigation into two species of amphibians. Both species (*Leptodactylus troglodytes* and *Rhinella granulosa*) occur throughout northeastern Brazil in the xeric Caatinga biome, which is bordered by savannahs to the west (the Cerrado biome) and rainforest to the east (the Atlantic Forest biome). While the individual distributions of these species differ slightly, with *L*. *troglodytes* widespread across the Caatinga and enclaves of this vegetation within the Cerrado and *R*. *granulosa* spanning the Caatinga and the Atlantic Forest, both species have been the subject of recent investigation with genomic data, with approximately 15,000 SNP for *L*. *troglodytes* [28] and approximately 7,000 SNP for *R*. *granulosa* [29]. In both species demographic model selection was used to detect changes in population sizes (either instantaneous or exponential) as an important component of the demographic history of each species. These systems were chosen because there are existing SNP data that provide evidence for the importance of population size change in the demographic history of each species and because mitochondrial DNA were not included in the original investigations. In addition to analyzing data from these species, we include analysis of published data from eight other taxa.

## Material and methods

### P2C2M.Skyline package

**Fig 1:**
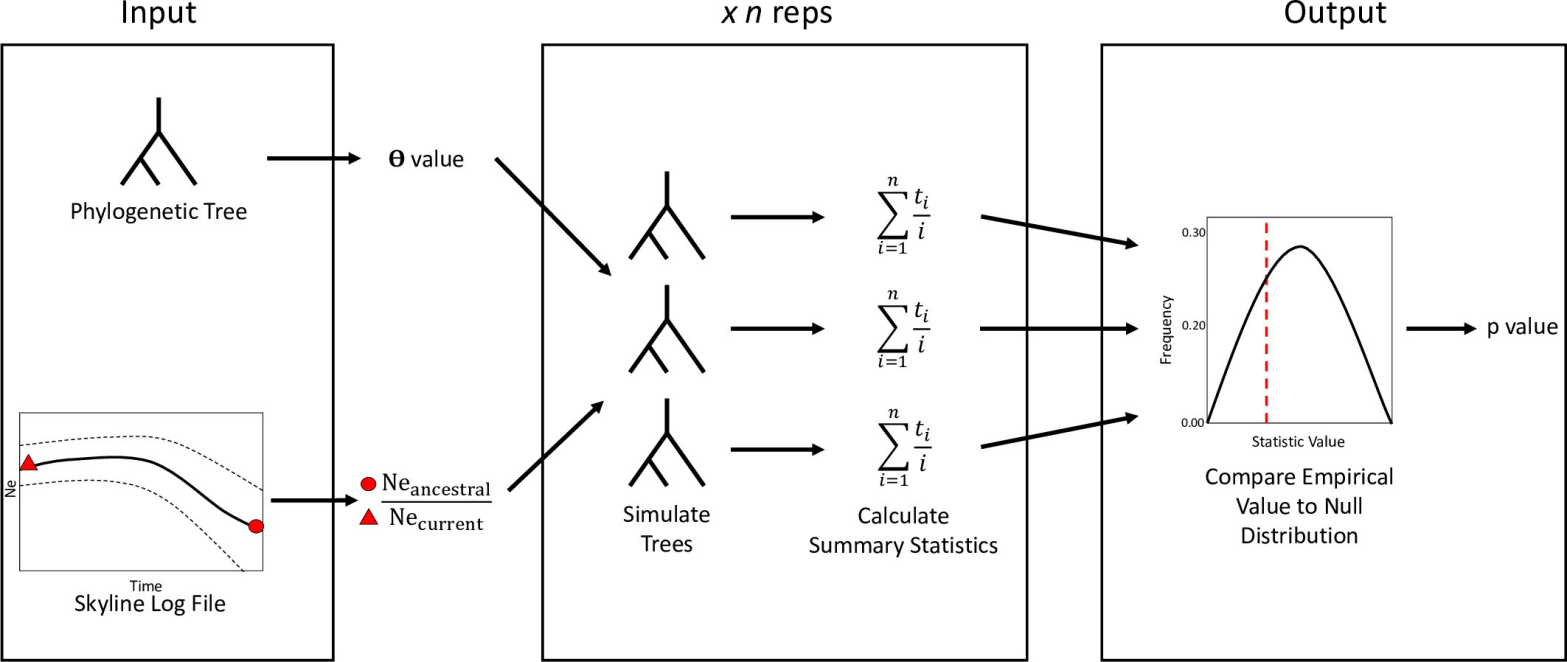
Workflow of the P2C2M.Skyline pipeline. Arrows represent the path of the data from step 1 to 6. See P2C2M.Skyline package section on Material and Methods for more information.

P2C2M.Skyline is an open-source R package designed to assess the statistical fit of the BSP model. The package is available at: https://github.com/P2C2M. User input to P2C2M.Skyline includes a phylogenetic tree and the log file resulting from a BSP analysis. The package check model fit to BSP model using PPS following Lewis et al. (2014). The general workflow is shown in Fig 1.

1. P2C2M.Skyline requires two input from users:
  - A ultrametric phylogenetic tree
  - The log file resulting from a Bayesian Skyline analyzed in Tracer [30].
2. P2C2M.Skyline calculates a theta value from the phylogenetic tree using the function *theta*.*tree* implemented in the *pegas* R package [31].
3. P2C2M.Skyline calculates the magnitude of the population size change by sampling a random value for ancestral and current population size from the credible interval from the posterior distribution of the Bayesian Skyline analysis (i.e., by choosing a value from the confidence interval). While the ancestral population represents the value of population size in the credible interval from the posterior distribution associate with the oldest coalescent time, the current population represents the value associate with the most recent time. Then, by dividing the ancestral population size by the current population size, the package calculates the population size ratio. This value is used in downstream analyses to model the population size trajectory through time (i.e., constant, expansion, or bottleneck).
4. P2C2M. Skyline uses the software ms [32] to simulate gene trees under a coalescent model. Three parameters are used to simulate the data: (i) the number of individuals from the empirical dataset; (ii) the q value calculated in step 2; (iii) the magnitude of the population size change calculated in step 3. The coalescent simulations are replicated “*n*” times, with the default value set to 100.
5. P2C2M.Skyline calculates for each simulated gene tree and the user supplied ultrametric tree a summary statistic: the sum of the cumulative coalescent interval divided by the reverse number of elements:
$$\sum_{i=1}^{n}\frac{t_{i}}{i}$$ (1)
  where *n* is the number of divergence events, *i* is ordered from the oldest (1) to youngest (n) divergence event, and *t*_*i*_ is the time of the *i*th divergence event. The summary statistic is calculated from all simulated datasets to construct the null distribution. Next, the summary statistic is calculated for the empirical dataset.
6. P2C2M.Skyline assesses the statistical fit of the Bayesian Skyline plot by calculating the number of simulated summary statistic values falling above and below the empirical value and then, multiplying the lesser value by two, which is equivalent to a two-tailed test. Next, a *p*-value is calculated by dividing this number by the total number of elements in the null distribution. A poor fit to the Bayesian Skyline is inferred if the *p*-value is lower than a user-defined threshold *α* (see Results).

### Simulation testing

To evaluate the performance of our package, we simulated datasets under different demographic histories and sampling schemes. Specifically, we used *ms* to simulate five evolutionary histories: (i) constant population size through time, (ii) population expansion, (ii) population bottleneck, (iv) two populations with shallow divergence, and (v) two populations with deep divergence. Because populations in (iv) and (v) are analyzed as a single population, these last two evolutionary scenarios represent models that violate the assumptions of the Bayesian Skyline model. Each evolutionary scenario was simulated under two sampling schemes: (i) 10 individuals and (ii) 50 individuals. For the two-population model, the number of individuals was equally distributed between the populations (i.e., each population had 5 or 25 individuals). We assumed a generation time between 3–5 years and an effective population size between 10,000–100,000 individuals, which is in the range of that observed in empirical systems (e.g., [20]). For the two-population models, we assumed a divergence time of 4N (shallow) and 8N (deep) generations in the past. We simulated datasets assuming a total of 200 segregating sites on a gene 1,000 bp long, totaling 1,000 datasets (100 replicates for each model under two distinct sampling schemes). DNA sequences of 1,000 bp were generated for each gene tree using Seq‐Gen [33] under the HKY model. We reconstruct for each dataset changes in population size through time using the Bayesian Skyline implemented in Beast2 (source code version; [34]). We used a strict molecular clock and ran the chain for 10^7^ generations, sampling every 10^3^ generations. We evaluated convergence using Tracer v1.7.1, ensuring the effective sample size was higher than 200 for all parameters. Then, Bayesian Skyline log files were analyzed using Tracer [30]. Gene trees were summarized using the maximum clade credibility tree in TreeAnnotator 1.8.0 [35]. Finally, we used P2C2M.Skyline assess the statistical fit of the BSP model to each simulated dataset. We evaluated the performance of P2C2M.Skyline under four significance values (1%, 2.5%, 5%, and 10%) using the Mathews Correlation Coefficient (MCC; [36]) implemented in the R package *mltools* [37].

### Summary statistics

We assessed the effectiveness of two additional summary statistics: (i) interval lengths [38] and (ii) summed branching times. While the former summary statistic is defined as the summed differences between time-interval lengths, the latter is the sum of the distance from each node to the tips. We used the simulated datasets to test the performance of both summary statistics in comparison to the summary statistic proposed previously.

### Applying P2C2M.Skyline to empirical data

We further assessed the utility of P2C2M.Skyline in 10 empirical datasets, consisting of mitochondrial DNA (Anura (4), Squamata (2), Passeriformes (3), and Araneae (1)). While most of the sequences were download from Genbank, we generated fragments of mitochondrial DNA for two of these empirical systems: *Rhinella granulosa* and *Leptodactylus troglodytes*. All sequences for both species are deposited in GenBank (accession numbers: numbers will be included upon acceptance). For these species, we extracted total DNA from liver and muscle preserved in ethanol using DNeasy Blood & Tissue kits (Qiagen, Venlo, Netherlands) and sampled the mitochondrial genome by sequencing the cytochrome oxidase subunit one (CO1) gene using the protocol and primers described in Lyra et al. [39]. Recent investigations (Thomé et al., [28]; Thomé et al., [29]), using genomic data, detected two populations for *R*. *granulosa* and three populations for *L*. *troglodytes*, respectively. Recent demographic changes were also detected for populations of both species based on phylogeographic model selection. In particular, population 3 of *L*. *troglodytes* showed a recent bottleneck, and the remaining populations showed signals of recent expansions. Because of this dynamic evolutionary history, both species represent excellent candidates to test P2C2M.skyline.

For all empirical datasets, we first selected the best model of nucleotide substitution using the Bayesian information criterion (BIC) implemented in JModelTest 2.0 [40]. Next, input files for P2C2M.Skyline were generated running BSP analysis as described for the simulated datasets. Empirical datasets were analyzed by grouping all the samples and by splitting them into different lineages as recovered in the original papers. We used a *α* value of 5% (see the Results of the simulations). We then compared the results of our analyses to those reported in the papers that described these data, where applicable. Our goal here was to assess the extent to which potentially misleading inferences result from cases of poor model fit.

## Results

### Simulation testing

**Fig 2:**
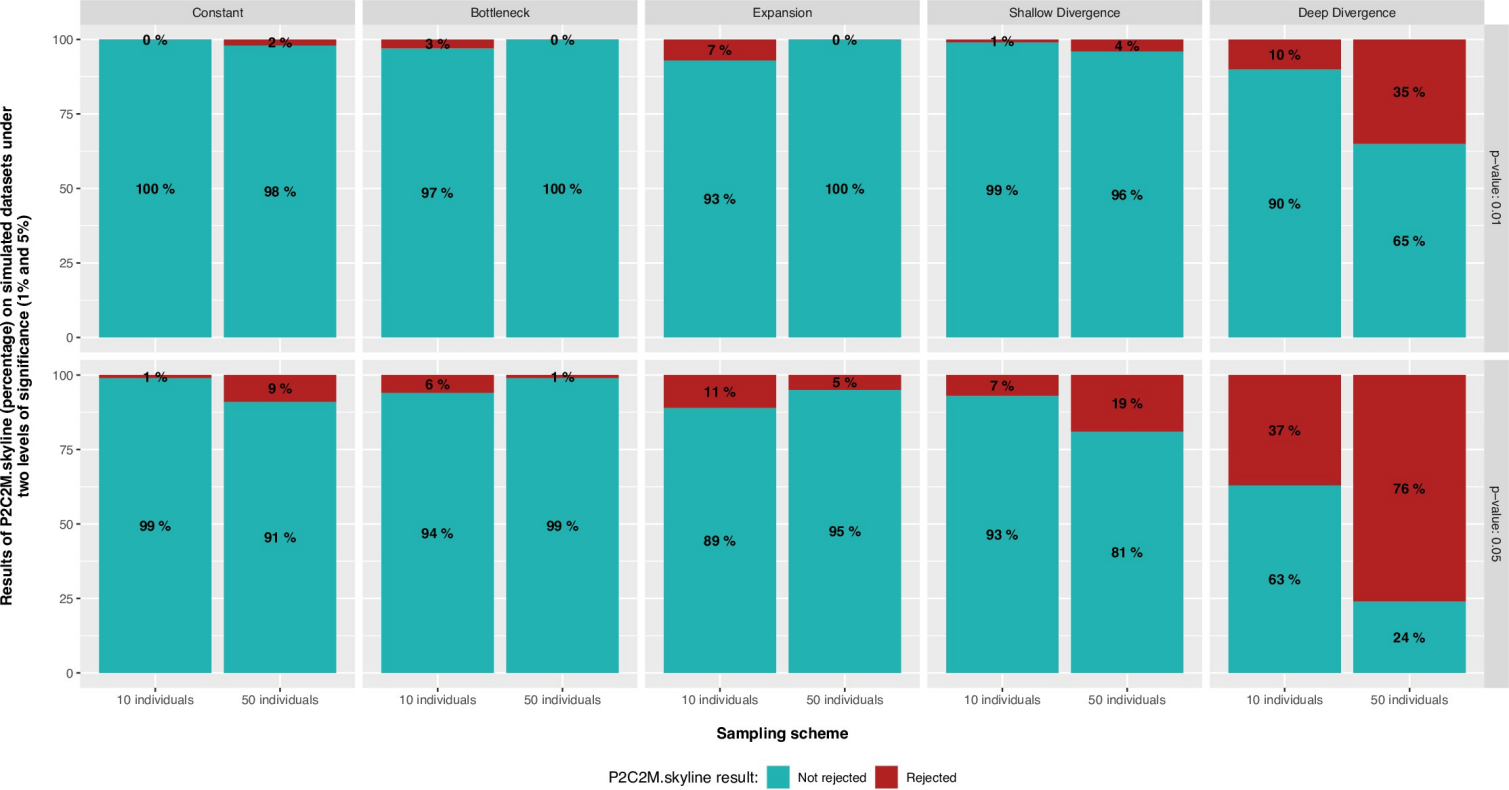
Percentage of simulated datasets with a p‐value of <1% and 5% across the five different diversification scenarios. In each chart, the Y‐axis shows the percentage of replicates where the statistical fit of the Bayesian Skyline model is rejected or not under two sampling schemes (10 and 50 individuals).

When there were no model violations (constant, bottleneck, and expansion datasets), P2C2M.Skyline failed to reject the Bayesian Skyline model for nearly all simulated datasets. (Fig 2 and S1 Fig in S1 File for *α* = 0.025 and 0.1). In contrast, for the two-population model (shallow and deep divergence), our package showed that many of the simulated datasets violate the Bayesian Skyline model (Fig 2 and S1 Fig in S1 File). The Matthews correlation coefficient (MCC) showed that the *α* value of 2.5% produced better results when compared to thresholds of 1%, 5%, and 10% (Table 1). However, this threshold also produced a high rate of false positives (i.e., datasets simulated under the correct premises that P2C2M.Skyline classified as a model violation). We advise users to use a threshold of 5% in their investigations because the lower rate of false negatives and reasonable values of MCC.

**Table 1:** Results of the mathews correlation coefficient for the simulated datasets. False positives represent datasets simulated under the Bayesian Skyline model premises (i.e., constant, expansion, and bottleneck) that P2C2M.Skyline classified as a model violation. In contrast, false negatives represent datasets not simulated under Bayesian Skyline model premises (i.e., two-population models) that P2C2M.Skyline classified as not a model violation.

Sampling	Significance level	True positives	Tue negatives	False negatives	False positives	Matthews correlation coefficient (MCC)
10 and 50 individuals combined	< 0.01	588	50	350	12	0.21
	< 0.025	583	99	301	17	0.33
	< 0.05	567	139	261	33	0.38
	< 0.1	523	245	155	77	0.50
10 individuals	< 0.01	290	11	189	10	0.05
	< 0.025	287	25	175	13	0.15
	< 0.05	282	44	156	18	0.23
	< 0.1	259	101	99	41	0.40
50 individuals	< 0.01	298	39	161	2	0.33
	< 0.025	296	74	126	4	0.48
	< 0.05	285	95	105	15	0.50
	< 0.1	264	144	56	36	0.61

### Summary statistics

We used MCC to assess the performance of interval lengths and summed branching times in comparison to the summary statistic proposed in step 5 (see P2C2M.Skyline package section). MCC under significance value of 5%, which showed to be the best significance value, showed that both summary statistics performed poorly compared to our proposed summary statistic. We recovered a MCC value of -0.14, 0.36, and 0.45 for interval lengths, summed branching times, and our proposed summary statistic, respectively. Given these results, which show that the proposed summary statistic maximizes true positive and true negative while minimizes false positives and false negatives, only this summary statistic is discussed further in the text. Information on each summary statistic is presented in S1–S3 Tables in S1 File.

### Applying P2C2M.Skyline to empirical data

Overall, our package performed well when applied to empirical datasets. For example, we identified model violations in five of eight systems when all samples were considered to belong to a single population (Table 2). In four of these cases, previous work identified population structure. This lumping of samples, which is a clear violation of the P2C2M.Skyline model, is obvious in retrospect in these cases due to other analyses conducted by the researchers, but since the characterization of population genetic structure is often a goal of investigations into empirical systems this may not be known during the initial stages of data analysis. For three of the eight datasets, despite evidence of population structure based on other molecular markers in previous studies, P2C2M.Skyline did not detect a model violation when all populations were lumped together.

**Table 2:** Results of the P2C2M.Skyline on empirical datasets. Asterisk indicates datasets with *p*-value < 0.05.

Order	Species	Population	Number of samples	Length (bp)	p-value	Skyline result	Reference
Anura	*Leptodactylus troglodytes*	All samples	82	641	0.46	Expansion	This study
		Population 1	32		0.12	Constant	
		Population 2	27		0.46	Constant	
		Population 3	23		0.42	Constant	
Anura	*Rhinella granulosa*	All samples	86	554	0.02*	Constant	This study
		Population 1	23		0.22	Constant	
		Population 2	63		0.092	Constant	
Anura	*Pleurodema alium*	All samples	25	603	0.044*	Constant	[41]
Anura	*Pleurodema diplolister*	All samples	165	603	0*	Expansion	[41]
		Population 1	15		0.006*	Constant	
		Population 2	140		0.968	Expansion	
		Population 3	10		0.074	Constant	
Squamata	*Polychrus acutirostris*	All samples	68	837	0.986	Constant	[18, 42]
		Population 1	18		0.176	Constant	
		Population 2	32		0.774	Constant	
		Population 3	18		0.482	Constant	
Squamata	*Lygodactylus klugei*	All samples	53	679	0.002*	Expansion	[18, 43]
		Population 1	45		0.146	Constant	
		Population 2	8		0.196	Constant	
Passeriformes	*Myrmeciza loricata*	All samples	44	1,041	0.07	Constant	[44]
Passeriformes	*Myrmeciza squamosa*	All samples	40	1,041	0.088	Constant	[44]
Passeriformes	*Myrmeciza loricate*	All samples	84	1,041	0.01*	Expansion	[44]
	+						
	*Myrmeciza squamosa*						
Araneae	*Sicarius cariri*	All samples	203	715	0.41	Expansion	[45]
		Population 1	162		0.028*	Expansion	
		Population 2	41		0.036*	Expansion	

When data from systems were analyzed on a population basis, P2C2M.skyline was not able to reject the Bayesian Skyline model for six of the eight of the datasets (Table 2). However, some empirical datasets (e.g., population 1 of *Pleurodema diplolister*) violated the Bayesian Skyline model even after samples were divided into populations (see Discussion for putative explanations). Interestingly, while P2C2M.Skyline did not detect a model violation when data from *Sicarius cariri* were analyzed as a single population, it did detect a model violation when data were analyzed separately for the two populations. P2C2M.Skyline took less than 1 minute to run in an average laptop (2.6 GHz Intel Core i5, 8 GB RAM) with a dataset composed of 100 DNA sequences. The skyline plots and the phylogenetic tree for each species are shown in S2–S10 Figs in S1 File.

## Discussion

### P2C2M.Skyline as a useful tool for empiricists

Bayesian Skyline plots are a commonly applied method for inferring the demographic history of populations. For example, they have been applied to characterize the trajectory of population size change in phylogeographic investigations (e.g., Table 2) and are also commonly used in epidemiology (e.g., [11, 46]). However, they make implicit assumptions about the conditions under which the data were sampled, and previous results have demonstrated that the inferences drawn from Bayesian Skyline plot analyses may be incorrect when the underlying assumption of an idealized Wright-Fisher population is violated (e.g., [47]). We developed a fast and friendly approach that applies PPS to evaluate model adequacy under the Bayesian Skyline model. Because it uses information that is available to all researchers who conduct Bayesian Skyline analyses, P2C2M.Skyline can be easily incorporated into the research pipeline and provide some assurance in the inferences that are drawn from Bayesian Skyline analyses. We follow Duchene et al. [11] in advocating that empiricists explore the model adequacy of the skyline plot model as part of the inference process. P2C2M.Skyline is easily incorporated into R analytical pipelines, making it suitable for automated phylogeographic analyses of thousands of datasets.

Our results demonstrate that P2C2M.Skyline is a powerful and fast tool for detecting model violations in Bayesian Skyline analyses. In general, we observed low numbers of false positives across various sample sizes and demographic histories. The ability to detect model violations under population structure scenarios was dependent on both the divergence time between the populations and the number of samples analyzed, with shallow divergence times and smaller sample sizes leading to more false negatives. Since deeper levels of population divergence are expected to have a larger effect on inferences drawn from Bayesian Skyline analyses [47], we believe this result to be ideal; the more extreme the model violation, the more likely P2C2M.Skyline is to detect the violation. We recommend using a conservative *α* of 0.05 to reduce false negatives, so that researchers are more likely to detect less-extreme model violations that may nevertheless mislead inference, particularly if a small number of samples is available. When a model violation is detected, we recommend that researchers analyze population structure and subsequently divide their dataset by population before performing skyline analyses again. Our empirical analyses show this to be an effective strategy for overcoming model violations (Table 2). Of course, our method may detect violations other than population substructure that were not evaluated here, such as migration. Thus, if dividing datasets into subpopulations still results in model violations, users may want to consider using tools other than BSPs to infer the demographic histories of populations. Overall, our results indicate that users of BSPs would benefit from incorporating P2C2M.Skyline into their workflow due to its fast run times and ability to detect model violations that may otherwise mislead inferences of population size changes.

Our tests of the P2C2M.Skyline pipeline used the cumulative coalescent interval as the summary statistic to determine if empirical datasets significantly differed from simulated ones, indicating model violations. This summary statistic is effective because it relies on distortions in branch lengths caused by population structure. Although we only examined population structure as a model violation, other model violations like selection or migration could also result in distorted branch lengths. Therefore, it is possible that our method could detect model violations other than just population structure. Further, other summary statistics may be powerful at detecting other, untested, model violations. Future research examining other model violations and other summary statistics could further improve the P2C2M.Skyline framework.

### Demographic history in the empirical datasets

Our analysis of the empirical data sets illustrates how P2C2M.Skyline can be of use when applied to empirical systems. For *L*. *troglodytes*, populations were analyzed individually by Thomé et al. [28], and findings included two populations that expanded in the late Pleistocene, and one population that endured an intense late Holocene bottleneck. Results from our BSP differed from this previous work, which can be attributed in large part due ot the mitochondrial data having less signal than the SNPs used by the previous study. However, the results of P2C2M.Skyline are clearly consistent with previous results, and had the mitochondrial data been analyzed first these results would have served as a useful guide to additional data collection in this system. For *R*. *granulosa*, the P2C2M.Skyline results demonstrate that the underlying model is not appropriate for the data when all samples are (incorrectly) combined into a single population. When samples are analyzed in the populations used by Thomé et al. [29], P2C2M.Skyline results are consistent with the findings from the analysis of SNP data, where the best model included some variation in population sizes related to an (smaller) ancestral population.

Results from the analysis of empirical data collected for other species highlight the utility of P2C2M.Skyline. As in *Leptodactylus troglodytes*, we did not detect a model violation when populations of *Polychrus acutirostris* were analysed as a single population. In both cases, population structure was determined based on nuclear and mitochondrial data, while the BSP analysis only considers mitochondrial data, perhaps explaining the inconsistencies. On the other hand, for *Pleurodema diplolister*, population structure was determined based on nuclear DNA, but our analyses still detected a model violation despite only considering mitochondrial data. Although use of only a mitochondrial marker may reduce the ability to reconstruct complex evolutionary scenarios because of the high stochastic variance associated with only one marker [25, 48], mtDNA markers are still commonly applied as a first pass for inference into the drivers of intraspecific diversification, especially if analyzed at the community-level [13, 18, 49]. Thus, attention should be drawn to population structure at this particular type of marker.

Finally, in two cases, we detect model violations even when population structure is taken into account. First, in population 1 of *P*. *diplolister*, we detect a model violation. Thomé et al. [41] did report *P*. *alium* mitochondria introgressing into population 1 of *P*. *diplolister*, which could explain the violation of the BSP model in this case. Similarly, for *Sicarius cariri*, we did not detect a model violation when populations were analyzed together but did detect model violations when the two populations were analyzed separately. This could be another case in which introgression from another population leads to a model violation and highlights the nuances inherent to determining what units to use when performing BSP analyses as well as the advantages of applying PPS to this problem.

## Conclusions

Here we develop a R package for assessing model adequacy for Bayesian Skyline plots using posterior predictive simulation. The package was successfully tested on simulated and empirical datasets. P2C2M.Skyline can be a useful tool for researchers interested in repurposing single locus phylogeographic data to address new questions using hierarchical ABC [13, 18], automated phylogeography (e.g., [20]), or predictive phylogeography (e.g., [21]).

## Supporting information

S1 File (DOCX)
