# Supplementary material for: Assessing model adequacy for Bayesian Skyline plots using posterior predictive simulation
Source: PLoS One. 2022 Jul 25;17(7):e0269438. doi: 10.1371/journal.pone.0269438 (PMC9312427; doi:10.1371/journal.pone.0269438)
Supplement: S1 File — (DOCX) [file pone.0269438.s001.docx]

##

**Assessing model adequacy for Bayesian Skyline Plots using posterior predictive simulation**

Emanuel M. Fonseca^1,2^, Drew J. Duckett^1,2^, Filipe G. Almeida^3^, Megan L. Smith^4,^ Maria Tereza C. Thomé^1,2^, Bryan C. Carstens^1,2^

^1^ Department of Evolution, Ecology and Organismal Biology, The Ohio State University, 318 W. 12th Ave., Columbus, OH, USA. 43210

^2^ Museum of Biological Diversity, The Ohio State University, 1315 Kinnear Rd., Columbus, OH, USA, 43212

^3^ Department of Zoology, Federal University at Juiz de Fora, Juiz de Fora, 36036-900, Minas Gerais, Brazil

^4^ Department of Biology and Department of Computer Science, Indiana University, Bloomington, IN 47405, USA

*Corresponding author: carstens.12@osu.edu; Department of Evolution, Ecology and Organismal Biology, The Ohio State University, 318 W. 12th Ave, Columbus, OH 43210

##

**Figure S1.** Percentage of simulated datasets with a p‐value of <2.5% and 10% across the five different diversification scenarios. In each chart, the Y‐axis shows the percentage of replicates where the statistical fit of the Bayesian Skyline model is rejected or not under two sampling schemes (10 and 50 individuals).

**Figure S2.** Phylogenetic tree and Skyline Bayesian plot for three datasets: (a) *Leptodactylus troglodytes* (all samples); (b) *Leptodactylus troglodytes* (population 1); (c) *Leptodactylus troglodytes* (population 2). Confidence intervals are outlined in blue.

**Figure S3.** Phylogenetic tree and Skyline Bayesian plot for three datasets: (a) *Leptodactylus troglodytes* (population 3); (b) *Rhinella granulosa* (all samples); (c) *Rhinella granulosa* (population 1). Confidence intervals are outlined in blue.

**Figure S4.** Phylogenetic tree and Skyline Bayesian plot for three datasets: (a) *Rhinella granulosa* (population 2); (b) *Pleurodema alium* (all samples); (c) *Pleurodema diplolister* (all samples). Confidence intervals are outlined in blue.

**Figure S5.** Phylogenetic tree and Skyline Bayesian plot for three datasets: (a) *Pleurodema diplolister* (population 1); (b) *Pleurodema diplolister* (population 2); (c) *Pleurodema diplolister* (population 3). Confidence intervals are outlined in blue.

**Figure S6.** Phylogenetic tree and Skyline Bayesian plot for three datasets: (a) *Polychrus acutirostris* (all samples); (b) *Polychrus acutirostris* (population 1); (c) *Polychrus acutirostris* (population 2). Confidence intervals are outlined in blue.

**Figure S7.** Phylogenetic tree and Skyline Bayesian plot for three datasets: (a) *Polychrus acutirostris* (population 3); (b) *Lygodactylus klugei* (all samples); (c) *Lygodactylus klugei* (population 1). Confidence intervals are outlined in blue.

**Figure S8.** Phylogenetic tree and Skyline Bayesian plot for three datasets: (a) *Lygodactylus klugeis* (population 2); (b) *Myrmeciza loricata* (all samples); (c) *Myrmeciza squamosa* (all samples). Confidence intervals are outlined in blue.

**Figure S9.** Phylogenetic tree and Skyline Bayesian plot for three datasets: (a) *Myrmeciza loricate* + *Myrmeciza squamosa* (All samples); (b) *Sicarius cariri* (all samples); (c) *Sicarius cariri* (population 1). Confidence intervals are outlined in blue.

**Figure S10.** Phylogenetic tree and Skyline Bayesian plot for: (a) *Sicarius cariri* (population 2). Confidence intervals are outlined in blue.

**Table S1.** Percentage of simulated datasets with a p‐value greater or lesser than 5% across the five different diversification scenarios using the proposed summary statistic in P2C2M.Skyline package section (see Material and Methods). Numbers show the percentage of replicates where the statistical fit of the Bayesian Skyline model is rejected or not under two sampling schemes (10 and 50 individuals).

| **Model** | **Sampling** | | | |
| --- | --- | --- | --- | --- |
|  | **10 individuals**  **(p > 0.05)** | **10 individuals (p < 0.05)** | **50 individuals (p > 0.05)** | **50 individuals (p < 0.05)** |
| Constant | 99 | 1 | 97 | 3 |
| Bottleneck | 91 | 9 | 91 | 9 |
| Expansion | 87 | 13 | 97 | 3 |
| Shallow Divergence | 45 | 55 | 29 | 71 |
| Deep Divergence | 83 | 17 | 69 | 31 |

**Table S2.** Percentage of simulated datasets with a p‐value greater or lesser than 5% across the five different diversification scenarios using interval lengths. Numbers show the percentage of replicates where the statistical fit of the Bayesian Skyline model is rejected or not under two sampling schemes (10 and 50 individuals).

| **Model** | **Sampling** | | | |
| --- | --- | --- | --- | --- |
|  | **10 individuals**  **(p > 0.05)** | **10 individuals (p < 0.05)** | **50 individuals (p > 0.05)** | **50 individuals (p < 0.05)** |
| Constant | 97 | 3 | 95 | 5 |
| Bottleneck | 91 | 9 | 93 | 7 |
| Expansion | 94 | 6 | 96 | 4 |
| Shallow Divergence | 100 | 0 | 100 | 0 |
| Deep Divergence | 99 | 1 | 99 | 1 |

**Table S3.** Percentage of simulated datasets with a p‐value greater or lesser than 5% across the five different diversification scenarios using summed branching times. Numbers show the percentage of replicates where the statistical fit of the Bayesian Skyline model is rejected or not under two sampling schemes (10 and 50 individuals).

| **Model** | **Sampling** | | | |
| --- | --- | --- | --- | --- |
|  | **10 individuals**  **(p > 0.05)** | **10 individuals (p < 0.05)** | **50 individuals (p > 0.05)** | **50 individuals (p < 0.05)** |
| Constant | 99 | 1 | 95 | 5 |
| Bottleneck | 92 | 8 | 84 | 16 |
| Expansion | 96 | 4 | 78 | 22 |
| Shallow Divergence | 48 | 52 | 30 | 70 |
| Deep Divergence | 90 | 10 | 76 | 24 |
